# A pilot effectiveness study of a just-in-time micro-randomized controlled trial on the physical activity and sedentary time of young children and their parents: The active family m-health intervention

**DOI:** 10.1371/journal.pone.0340687

**Published:** 2026-01-16

**Authors:** Sophie M. Phillips, Matthew Bourke, Bayley V. Inniss, Manvir Ahluwalia, Patricia Tucker

**Affiliations:** 1 Child Health and Physical Activity Laboratory, School of Occupational Therapy, Western University, London, Ontario, Canada; 2 Health and Wellbeing Centre for Research Innovation, School of Human Movement and Nutrition Sciences, The University of Queensland, Brisbane, Australia; 3 Health and Rehabilitation Sciences Program, Faculty of Health Sciences, Western University, London, Ontario, Canada; 4 Children’s Health Research Institute, London, Ontario, Canada; Faculty of Physical Education and Sports at Pedagogical University of Maputo, MOZAMBIQUE

## Abstract

**Background:**

Parents play a critical role in influencing their young children’s physical activity (PA) and sedentary time (ST). Despite this, many young children (aged 3-4y) and their parents are insufficiently active and engage in high amounts of ST. M-health interventions targeting PA and ST have seldom been tested in this population. The objective of this study was to examine the effectiveness and acceptability of the Active Family m-health intervention on the PA and ST of young children and their parents.

**Methods:**

Twenty-five stay-at-home parent-child dyads from Canada took part in the 2-week just-in-time micro-randomized controlled trial. Parents received seven text message prompts per day, where they were randomized to receive either a micro-intervention (activity suggestion) or control (no suggestion). Parents and children wore ActiGraph accelerometers to measure ST, light [LPA], and moderate-to-vigorous physical activity [MVPA]. Parents also completed a short online acceptability survey. A centred and weighted least square regression was used to analyze the effect of activity suggestions on the 60-min ST, LPA, and MVPA of parents and children following suggestion randomization. Descriptive statistics and content analysis were used to analyze acceptability survey responses.

**Results:**

Micro-interventions were not effective at changing children’s or parent’s proximal ST (d = 0.01, p = .878; d = −0.09, p = .485, respectively), LPA (d = 0.03, p = .714; d = 0.03, p = .729, respectively), or MVPA (d = −0.05, p = .511; d = 0.10, p = .480, respectively). Interventions became more effective at increasing MVPA over time for parents (b = 0.47, 95%CI = 0.12, 0.83, p = .013). Among children, intervention effectiveness varied by contextual factors (e.g., weather). The intervention was largely acceptable, appropriate, and feasible for parents, though they did offer suggestions for improvement.

**Conclusions:**

Overall, micro-interventions did not significantly change parents or young children’s proximal movement. Though, this approach showed promise for increasing parent’s MVPA over time and for supporting children’s activity under specific conditions.

## Introduction

Physical activity (PA) and sedentary time (ST) are associated with a range of physical, psychosocial, and cognitive outcomes for young children [1,2]. The World Health Organization recommends that young children (aged 3–4 years) should engage in 180 minutes of PA per day, of which 60 minutes should be moderate-to-vigorous PA (MVPA), and should limit sedentary screen-time to less than 1 hour per day [3]. Despite these recommendations, more than 40% of children aged 3–4 years in Canada do not meet current PA recommendations, and more than 75% do not meet sedentary screen time guidelines [4]. PA and ST have been shown to track from early childhood, highlighting the importance of intervening on these behaviours at a young age [5].

Parents play a primary role in the movement behaviours of their children, with consistent evidence showing a positive association between parent and child PA levels [6,7]. This association is strongest when children are in their early years (aged 0–6 years) [7], with recent data in Canada demonstrating that parental PA and ST are strongly correlated with those of their young children [8]. The mechanisms underlying parental influence on child PA are multifaceted and grounded in several behavioural theories. Consistent with social learning theories, parents may encourage PA in their children through parental modelling of PA behaviours [9], as well as through activity encouragement and support [10]. Through continual modelling and co-participation, parents may create habits where contextual cues become reliably related to PA behaviours, leading to active family routines where PA becomes autonomized as part of a parent and child’s habitual behaviours [11]. Beyond modelling and habit formation, Self-Determination Theory (SDT) provides a framework for understanding how parenting practices shape children’s intrinsic motivation for PA [12]. SDT suggests that parents who provide autonomy-supportive environments foster children’s autonomous motivation and sustained engagement in PA. Recent evidence from web-based interventions targeting need-supportive parenting behaviours demonstrates that training parents to satisfy children’s basic psychological needs (autonomy, competence, and relatedness) can significantly enhance children’s PA intentions and behaviours [13,14]. While this research has primarily focused on older children and adolescents, the theoretical principles of need-supportive parenting apply across developmental stages and may be particularly potent during early childhood when parental influence is strongest [7].

Parents and children engage in sedentary activities the majority of time they are together, spending less than 1% of their time together each day co-participating in MVPA [15,16]. Therefore, parent-focused interventions have potential to increase PA among young children. Indeed, interventions that target parent-child dyads may be more effective at increasing levels of PA and reducing ST, compared to interventions that target children alone [17]. Further, interventions that target parent-child dyads also have the potential to increase parents’ PA, and this dual benefit may contribute to sustained behaviour change in parents and their children over time [18]. As well, this has further advantage as PA levels of parents with preschoolers are reported to be lower than those with older children and those without children [19,20]. Only 8% of mothers and 10% of fathers with young children are reported to meet the PA guidelines of 150 minutes MVPA per week [19,21]. Notwithstanding, research has reported that parents find it difficult to be active with children and are uncertain on how to create an active family culture [22], which further emphasises the importance of providing support and intervention. Therefore, targeting parent-child dyads may be an effective strategy to increase young children’s engagement in PA and decrease ST.

As digital technologies, such as smartphones, laptops and tablets, have become more ubiquitous, there has been a rapid increase in the adoption of e-health interventions [23]. E-health refers to the wide range of technologies used to facilitate delivery of health care [24]. There has been rapid growth in the implementation of PA and ST interventions using e-health approaches [25]. While e-health interventions show promise for improving PA in adults [26,27], there are conflicting findings regarding their effectiveness for improving PA levels of young children [28]. Though, a recent meta-analysis demonstrated that when compared to control groups, e-health interventions significantly increased total PA, MVPA, and reduced ST of young children [29], suggesting that certain interventions using e-health approaches may show promise for improving activity levels of young children.

M-health interventions are a specific type of e-health intervention that use mobile phones or other mobile devices (i.e., personal digital assistants) as their delivery platform [30]. These interventions have been shown to be particularly effective at improving PA behaviours in older children and adults [31–33]. However, despite their efficacy in other populations [34], m-health interventions targeting PA and ST have rarely been tested in young children and their parents, with minimal research examining the effectiveness and acceptability of such an intervention approach in this cohort [35,36]. Those that have include the MINISTOP trial, consisting of an m-heath phone application aimed at improving parental knowledge, skills, and self-efficacy to support diet and PA behaviours of their young children, which has yielded minimal effectiveness on PA of young children [35,37,38]. Further, the Mini Movers intervention, predominantly a text-message delivered intervention, provides parents with support to minimize sedentary and screen time, which demonstrated statistically significant reductions in children’s screen time [36]. Given the potential for effectiveness of certain m-health interventions, the omnipresence of mobile phones, alongside parental reports of the use of mobile devices for family routines, behaviour management, and support of these devices for intervention delivery [39] – there is an opportunity to harness the potential of m-health interventions to improve activity levels of young children and their parents.

One novel approach to deliver m-health interventions, which has not yet been explored in young children, is a *Just-In-Time Adaptive Intervention* (JITAI; [40]). JITAIs recognize the dynamic nature of human behaviour and aim to deliver support when people need it most and are likely to be receptive to the intervention [40]. JITAIs use proximal tailoring variables and decision rules to offer one of an array of micro-intervention options that is likely to have the most beneficial outcome at the moment the intervention is delivered [41]. JITAI approaches are thought to be especially useful for interventions to increase PA and reduce ST, given their responsiveness to changing contexts, such as environmental factors, which can moderate the effectiveness of interventions targeting these behaviours [42]. The aim of JITAIs is to deliver micro-interventions to users multiple times each day, which are specifically tailored to their momentary behaviours and contexts. For example, if participants report being sedentary watching television at home while it is raining outside at a ‘decision point’ (i.e., when they receive an intervention text message), a micro-intervention tailored towards breaking up ST with PA around the house will be delivered. Alternatively, if participants report that it is sunny outside, a micro-intervention that aims to get the participant active outdoors may be delivered. The aim of JITAIs is to influence proximal behaviours. Although the effectiveness of JITAIs for PA in adult populations is apparent [43–45], the effectiveness and acceptability among parent-child dyads is less clear [46]. Nevertheless, the transactional nature of parent-child relationships means that behavioural opportunities and barriers often emerge simultaneously. Parents and young children share proximal contexts creating synchronous windows of opportunity where intervention prompts can target both members of the dyad when conditions are optimal. Additionally, given that parents play a vital role in supporting their children to be physically active [10], JITAIs may encourage active motivation and participation among parents and their children when behavioural interventions are most likely to be effective.

As such, the purpose of the current study was to examine the acceptability and proximal effectiveness of the Active Family m-health intervention, a contextually tailored JITAI, on the PA and ST of parent-child dyads. Additionally, the study aimed to explore under what conditions the JITAI may be most effective at increasing engagement in PA and reducing ST among children and their parents.

## Methods

### Study design and procedures

**Fig 1:**
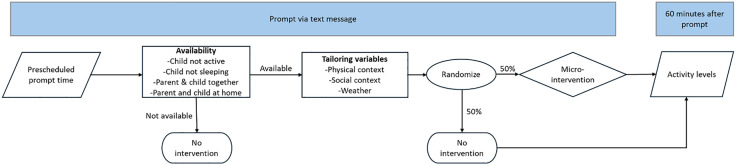
Schematic of the Active Family micro-randomized controlled trial.

A micro-randomized controlled trial design was used for this study, to examine the proximal causal effects of the JITAI on children’s and their parent’s PA and ST [47,48]. Specifically, at each decision point (i.e., each text prompt), parent-child dyads were randomized to receive a micro-intervention or control condition. Differences in proximal behaviours (e.g., in 60 minutes immediately after each decision point) are compared within participants over the course of the study period to determine if the JITAI impacts proximal outcomes [38]. An overview of the micro-randomized controlled trial is shown in Fig 1.

First, parents were sent an ecological momentary assessment (EMA) prompt via a text message delivered using an online text messaging service (https://app.textmagic.com) with a link to a Qualtrics questionnaire. A total of 7 prompts were sent each day, at random times and at least 60 minutes apart from each other, with a total of 98 text messages sent over the 2-week intervention period. Before the intervention, parents were asked to provide information on when they were unavailable (e.g., due to other commitments) over the 2-week period, to ensure text messages were sent when it was likely that parents and their children were available. Participants were randomly assigned to one of four pre-generated prompting schedules using an online random number generator. Each prompting schedule delivered text messages between 7am and 7 pm in the participant’s local time. Each prompt had an equal likelihood of being an intervention or control prompt. At each prompt, parents were sent a brief questionnaire with several questions to assess availability (see S1 File). These availability questions asked if parents were with their child, if their child was awake, if they were at home, and what their child was doing (i.e., sitting down, standing around, or being active). If parents responded that they were not with their child, their child was asleep, they were not at home, and/or their child was already active, they were considered unavailable and no further tailoring variable questions were asked and no micro-intervention was delivered. If it was determined that the parent and child were available for an intervention, additional tailoring variables were measured, including if the parent and child were inside or outside, who else they were with, and what the weather was like. The intervention text content was immediately present upon completion of the survey. Responses completed within 60 minutes of the text message being sent were considered valid and included in the analysis. Responses completed outside 60 minutes were excluded and considered missing.

**Fig 2:**
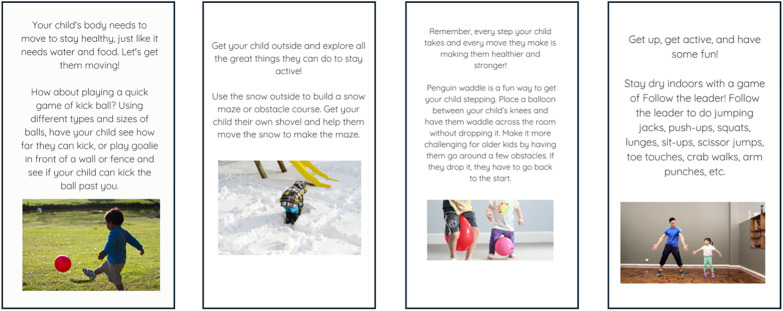
Examples of micro-intervention content delivered to parents.

Each micro-intervention included an activity recommendation that acted as a cue for parents and their children to be active. Recommendations were based on parents’ responses to tailoring variables and contained factors from the Fogg Behaviour Model [49]: motivation, ability, and triggers. A suggestion acted as a trigger and contained elements to motivate behaviour change (e.g., getting moving is fun) and increase ability (i.e., by providing an appropriate recommendation). Consistent with the SDT, micro-interventions were designed to enhance parental competence by providing contextually feasible, age-appropriate activities matched to reported circumstances (e.g., indoor options during rain, brief activities when time-limited). Prompts emphasized co-participation to foster parent-child relatedness and enable parents to model PA behaviours for their children. An array of intervention content developed by the research team was available for each possible combination of answers and included activity suggestions, embedded videos from the internet, and links to publicly available activity resources. A total of 73 micro interventions were developed, with between 7–37 micro-interventions available for every unique combination of contextual tailoring variables. Examples of micro intervention content are displayed in Fig 2. Random intervention content applicable based on parents’ responses to tailoring variables was selected using branches and randomizers in a Qualtrics survey flow (https://www.qualtrics.com/). The selection of a micro-intervention at each prompt was independent of previous prompts, meaning that micro-intervention content could be repeated over the course of the intervention. When randomized to a control prompt, parents completed identical availability and tailoring questions but were not provided with any micro-intervention content.

Recruitment and data collection took place over the course of one year between April 2024 and May 2025. At baseline, parents completed a demographic questionnaire. Parents and children were then mailed accelerometers which they were asked to wear for the duration of the two-week intervention to measure their ST and PA. At the end of the intervention, parents completed an online questionnaire to assess acceptability of the intervention. Families were compensated in gift cards for their participation in the study.

### Ethics

Ethical approval was provided by the Health Sciences Research Ethics Board at Western University in London, Ontario, Canada (REB# 123093). Parents and guardians were provided with information about the study prior to participating and consenting. Consent was provided by parents/guardians for themselves and their child by signing and returning a completed consent form to the research team.

### Participants

Stay-at-home parent-child dyads living in Canada were recruited using targeted posts placed on social media (i.e., Facebook, Instagram, Reddit, Twitter/X), as well as advertisements in libraries, recreation and family centres, and through word of mouth. Recruitment posters were available in both official languages in Canada (i.e., English and French), though only English-speaking parents were eligible to participate due to the intervention texts being available in this language. Parent-child dyads were eligible to participate if: a) their child was aged 3–4 years; b) the participating child was in childcare no more than one day each week (to ensure that they were with their parent for the majority of the intervention period); c) the parent was a stay-at-home parent with full custody of the child; d) the parent owned a mobile phone with access to the internet; e) was able to understand and respond to questionnaires written in English; and f) lived in Canada. Parent-child dyads were not eligible to participate in this study if their child had a physical disability which precluded them from engaging in physical activities (e.g., muscular dystrophy). Additionally, parent-child dyads that had lower than a 10% compliance rate on EMA prompts were excluded from the analysis.

### Sample size calculations

Sample size was calculated using the MRT-SS Calculator shiny app (https://d3center.shinyapps.io/mrt-ss-calculator/). The estimated required sample size assumed a small-to-moderate standardized average proximal treatment effect ($\overset{―}{d}$ = 0.20), 98 decision points (7 decision points per day for 14 days), and a participant availability of 50%. To achieve 80% power, with an alpha of 0.05, a sample size of 20 parent-child dyads was targeted. This sample was considered efficient to collect and analyse acceptability data from parents.

### Measures

#### Demographics.

Parents completed a baseline questionnaire reporting on demographic characteristics about their child, themselves, and their household prior to starting the intervention. Pertaining to their child, demographic characteristics included sex, gender identity, age, ethnicity, time spent in childcare, PA, enrolment in extra-curricular sports/activities, any physical, neurodevelopmental, or medical condition affecting their ability to play or be physically active, sleep problems (e.g., bed wetting, night terrors, sleep walking), and if they are suffering from an illness or are unwell, which is affecting their normal behaviour. Parents reported on their age, gender identity, relationship with the child, level of education, employment status, and PA. Household demographic characteristics included geographical location, family situation, and household yearly income.

#### Physical activity and sedentary time.

PA and ST were assessed using the ActiGraph wGT3x-BT accelerometers (Actigraph LLC, Fort Walton Beach, FL). Children wore the accelerometers on their right hip, whilst the participating parent wore the accelerometer on their non-dominant wrist for the duration of the 2-week intervention. Parent-child dyads were provided with the option to wear the accelerometers while sleeping; however, this was not mandatory. The ActiGraph accelerometer has been shown to be valid for the assessment of PA and ST of young children, when assessed in relation to reference methods of both calorimetry and direct observation [50], as well as adults, including being one of the most frequently used activity monitors in physical activity research [51]. Accelerometers were initialized and processed using ActiLife software (v6.13.4, Firmware 1.9.2). All accelerometer data were collected using a 30 Hz sampling frequency and downloaded using age and device-location specific processing decisions. The children’s accelerometer data were downloaded using a 15s-epoch and processed using Janssen cut-points [sedentary time 0–100 counts, LPA 101–1679, and MVPA >1680 counts per minute] [52,53] Parents accelerometer data were downloaded using a 60s-epoch and processed using Montoye cut-points [sedentary time 0–2859 counts, LPA 2860–3940, and MVPA >3941 counts per minute] [54,55]. Proximal outcomes were operationalized as the time spent in ST, LPA, and MVPA after receiving a micro-intervention or control. Additionally, the time spent in ST, LPA, and MVPA before receiving a micro-intervention was included in the model as a covariate. Non-wear time was classified as a period of at least 20 minutes of consecutive zero counts, for both adults and their children [56]. Accelerometer data was included if there was valid accelerometer data for at least 50% of the 60-minute window prior to and following a prompt, handled separately for child and parent data (i.e., if a child had valid data at a prompt but the parent didn’t the child’s data was still included). In total, 16% of the cases included in the child analysis had some missing accelerometer data in the 60 minutes following a prompt (average wear time = 45 minutes). For parents, 8% of cases included in the analysis had some missing data (average wear time = 44 minutes). To account for missing data, all accelerometer data was standardized to a total of 60 minutes following a micro-intervention.

#### Post-intervention acceptability questionnaire.

Following completion of the 2-week intervention period, parents were asked to complete a post-intervention questionnaire via Qualtrics to assess their satisfaction with the intervention, including perceived acceptability, appropriateness, and feasibility of the intervention. Parents were asked 3 questions devised for the purpose of this study on whether the intervention taught them something new about motivating their child to be active, as well as if they were able to use the intervention to help both their child and themselves be more active. Parents were also asked implementation outcome questions using a validated 12-item questionnaire that assesses acceptability, appropriateness, and feasibility of interventions [57]. Respondents were asked to rank each item on a 5-point Likert scale (1 = *Completely disagree* to *5 = Completely agree*). Finally, parents were asked 4 open-ended questions (i.e., likes, dislikes, suggestions for improvement, any other comments).

### Data analysis

Descriptive statistics were calculated for all demographic variables, as well as the post-intervention acceptability questionnaire. Open ended questions were analyzed using manifest content analysis with inductive coding in a deductive framework (i.e., analysis guided by the question prompts of likes, dislikes, suggestions, but within each category inductive coding to identify recurring themes) [58]. Compliance for EMA prompts was calculated, and changes in compliance over the course of the intervention was estimated using a generalized linear mixed effect model. Intervention effectiveness analyses were conducted using R v. 4.1.3 (R Core Team, Vienna, Austria) in R studio v. 1.3 (RStudio Team, Boston, MA). The effect of the micro-interventions on PA and ST was assessed using centred and weighted least square regression using the MRTAnalysis package [59]. The weighted and least squares regression estimates the average causal effect of receiving an intervention on PA and ST across the intervention [60]. Separate models were estimated for ST, LPA, and MVPA, for children and their parents. Further, whether the effect of micro-interventions changed over the intervention period were also explored by including an interaction term between intervention and day of the study. Finally, to see if the intervention effect differed based on contextual factors, models with interaction terms for physical environment (outdoors, indoors), social environment (alone, with other adults, with other children, with other children and adults), and weather (pleasant, too hot, too cold, raining/snowing) were estimated. To increase precision of the average causal effect, each model included levels of ST, LPA, or MVPA in the 60 minutes before the prompt as a time-varying covariate. A standardized effect size (Cohen’s d) was calculated by dividing the average causal effects by the standard deviation of the outcome.

## Results

A total of 25 parent-child dyads provided consent to participate in this study (see Table 1 for participant demographics). Four participants did not return the accelerometers, and of these, three did not complete the surveys. One participant was provided with an incorrect prompting schedule. Additionally, data was excluded for two parent-child dyads who completed fewer than 10% of sent prompts. As such, we had usable effectiveness data (i.e., intervention survey and accelerometer data) from 18 dyads (72%) and usable acceptability data (i.e., post-intervention acceptability survey) from 22 dyads (88%). Out of a possible 1764 EMA prompts, parents completed 1388 prompts (80% compliance rate). The compliance rate increased slightly over time (b = 0.11, p < .001). Parents and children were available at 783 (42%) decision points. When parent-dyads were not available to receive a micro-intervention, 52% of occasions it was due to not being at home, 24% of the time it was due to the child being asleep, and 24% of the time it was because the parent and child were not together. Due to missing accelerometer data, 510 points had valid data for children (65% of available prompts) and 481 decision points had valid data for parents (61% of available prompts).

**Table 1:** Descriptive Statistics for Participant Demographics.

Characteristic	*n* (%)
**Children**	
**Age** – Months, M(SD)	45.5 (8.65)
**Sex**	
Male	9 (36%)
Female	16 (64%)
**Gender**	
Boy	10 (40%)
Girl	15 (60%)
**Ethnicity**	
White	20 (80%)
South Asian	2 (8%)
Chinese	1 (4%)
Latin-America	1 (4%)
Japanese	2 (8%)
Indigenous	2 (8%)
Other	1 (4%)
**Hours per Week in Childcare**	
They do not attend childcare	20 (80%)
2-4 hours	1 (4%)
5-6 hours	3 (12%)
6-8 hours	1 (4%)
**Parental Reported Child PA**^**a**^ – Minutes, M(SD)	159.57 (112.5)
**Parental Reported Child High Intensity PA** – Minutes, M(SD)	56.79 (37.7)
**Extra-Curricular Sports/Activities**	
Yes	16 (64%)
< 2 hours	12 (75%)
2-5 hours	4 (25%)
No	9 (36%)
**Physical, Neurodevelopmental, or Medical Condition Affecting Ability to Play or be Physically Active**	
Yes	
No	0 (0%)
	25 (100%)
**Sleep Problems**	
Yes	5 (20%)
No	20 (80%)
**Suffering From Illness or Unwell Affecting Normal Behaviours**	
Yes	2 (8%)
No	23 (92%)
**Parents**	
**Age** – Years, M(SD)^a^	36.25 (5.8)
**Gender**	
Man	1 (4%)
Woman	24 (96%)
**Relationship with Child**	
Father	1 (4%)
Mother	24 (96%)
**Level of Education** ^**b**^	
Graduate School	3 (12%)
University	11 (44%)
College	6 (24%)
Secondary School	4 (16%)
Elementary School	1 (4%)
**Employment Status**	
Employed full-time	1 (4%)
Employed part-time	5 (20%)
Self-employed	1 (4%)
Casual employment	3 (12%)
Unemployed	8 (32%)
Other	7 (28%)
**Days in the past week with at least 30 min of leisure‐time MVPA**	
0 days	1 (4%)
1 day	1 (4%)
2 days	3 (12%)
3 days	3 (12%)
4 days	3 (12%)
5 days	8 (32%)
6 days	3 (12%)
7 days	3 (12%)
**Household**	
**Geographical Location**	
Alberta	5 (22%)
British Columbia	6 (26%)
Manitoba	1 (4%)
Newfoundland and Labrador	1 (4%)
Ontario	10 (44%)
Saskatchewan	1 (4%)
Missing	1 (4%)
**Family Situation**	
Double-parent	21 (84%)
Single-parent	1 (4%)
Other	2 (8%)
Prefer not to answer	1 (4%)
**Yearly Household Income**	
More than $150,000	2 (8%)
$120,000-$149,999	2 (8%)
$100,000-$119,999	4 (16%)
$80,000 - $99,999	2 (8%)
$60,000 - $79,999	4 (16%)
$40,000 - $59,999	3 (12%)
$20,000 - $39,999	3 (12%)
Less than $20,000	3 (12%)
Prefer not to answer	2 (8%)

^a^Four responses were removed from parents’ mean age, as assumed question was misread and provided their child’s age instead of their own age; PA = physical activity; MVPA = moderate-to-vigorous physical activity.

^b^In Canada, universities are post-secondary institutions authorized to grant academic degrees (e.g., bachelor’s, master’s, doctoral). Colleges are post-secondary institutions that offer diplomas, certificates, and applied or technical training.

### Effects of the active family intervention on ST, LPA, and MVPA of children and parents

#### Child outcomes.

On average, in the 60 minutes before all included prompts, children spent 30.3 minutes sedentary (SD = 12.3), 22.6 minutes in LPA (SD = 8.8), and 7.1 minutes in MVPA (SD = 6.7). On average, in the 60 minutes after all included prompts, children spent 29.8 minutes sedentary (SD = 12.1), 23.0 minutes in LPA (SD = 8.3), and 6.5 minutes in MVPA (SD = 6.8). The effects of providing contextually tailored activity suggestions on children’s and their parents’ ST, LPA, and MVPA are displayed in Table 2. For children, the micro-interventions were not effective at changing time spent sedentary (d = 0.01, *p* = .878), in LPA (d = 0.03, *p* = .714), or in MVPA (d = −0.05, *p* = .511). The effect of the intervention did not significantly change over time for ST (b = −0.07, 95%CI = −0.49, 0.34, *p* = .717), LPA (b = 0.16, 95%CI = −0.20, 0.51, *p* = .365), or MVPA (b = −0.10, −0.44, 0.23, *p* = .513) among children. Effect moderators are displayed in Table 3. The intervention was more effective at reducing ST when children were indoors at the time of the prompt (d = −0.36, *p* = .025), and it was more effective at increasing MVPA when children and their parent were not with other adults at the time of the prompt (d = 0.39, *p* = .003) and when the weather was pleasant compared to too cold (d = 0.34, *p* = .031).

**Table 2:** Average Causal Effect of Micro-Interventions.

	Average Causal Effect	95%CI
**Children**		
ST	0.13	−1.68, 1.95
LPA	0.23	−1.09, 1.56
MVPA	−0.36	−1.50, 0.78
**Parents**		
ST	−1.22	−4.47, 2.22
LPA	0.29	−1.46, 2.04
MVPA	0.79	−1.54, 3.14

Note. ST = Sedentary Time, LPA = Light Physical Activity, MVPA = moderate-to-vigorous physical activity.

**Table 3:** Moderator of the Average Causal Effect of Micro-Interventions.

	Sedentary	LPA	MVPA
**Children**			
**Physical environment**			
Indoors (ref)			
Outdoors	**4.34 (0.63, 8.05)**	−1.75 (−5.51, 2.01)	−2.40 (−6.41, 1.60)
**Social environment**			
Alone (ref)			
With other children	0.50 (−6.17, 7.16)	−0.27 (−4.95, 4.14)	0.12 (−2.35, 2.59)
With other adults	4.21 (−3.73, 12.16)	−1.51 (−8.30, 5.28)	**−2.67 (−4.16, −1.18)**
With adults and children	0.33 (−6.43, 7.10)	−0.40 (−5.55, 4.75)	0.29 (−2.61, 3.18)
**Weather**			
Pleasant (ref)			
Too hot	0.26 (−4.93, 5.44)	2.13 (−1.09, 5.35)	−2.13 (−5.89, 1.62)
Too cold	0.52 (−2.93, 3.96)	1.99 (−1.35,5.34)	**−2.31 (−4.36, −0.27)**
Raining/snowing	2.19 (−2.26, 6.65)	0.60 (−3.77, 4.97)	−2.52 (−5.29, 0.26)
**Parents**			
**Physical environment**			
Indoors (ref)			
Outdoors	−4.46 (−12.37, 3.45)	3.46 (−4.79, 11.72)	0.89 (−2.79, 4.57)
**Social environment**			
Alone (ref)			
With other children	−0.44 (−6.40, 5.53)	−1.22 (−5.76, 3.34)	1.17 (−3.49, 6.93)
With other adults	−2.95 (−18.37, 12.46)	−4.35 (−9.38, 0.68)	7.30 (−4.51, 19.11)
With adults and children	−0.65 (−5.97, 4.67)	−2.40 (−6.12, 1.32)	3.12 (−1.69, 7.93)
**Weather**			
Pleasant (ref)			
Too hot	−0.17 (−10.48, 10.14)	1.14 (−7.55, 9.82)	−0.97 (−5.33, 3.39)
Too cold	−2.16 (−13.73, 9.40)	0.14 (−5.10, 5.38)	2.01 (−5.23, 9.27)
Raining/snowing	1.03 (−9.33, 11.38)	−2.93 (−6.98, 1.12)	1.87 (−6.05, 9.80)

***Note:*** Significant outcomes are bolded. ST = Sedentary Time, LPA = Light physical activity, MVPA = moderate-to-vigorous physical activity.

#### Parent outcomes.

On average, in the 60 minutes before all included prompts, parents spent 34.8 minutes sedentary (SD = 13.1), 15.7 minutes in LPA (SD = 8.3), and 9.4 minutes in MVPA (SD = 8.2). Parents engaged in an average of 35.2 minutes of ST (SD = 13.1), 15.1 minutes of LPA (SD = 8.0), and 10.4 minutes of MVPA (SD = 9.0) in the 60 minutes following included prompts. The micro-interventions were not effective at changing parents’ time spent sedentary (d = −0.09, *p* = .485), in LPA (d = 0.03, *p* = .729), or in MVPA (d = 0.10, *p* = .480). The effects of the interventions did not significantly change over time for ST (b = −0.31, 95%CI = −1.00, 0.38, *p* = .347) or LPA (b = −0.15, 95%CI = −0.61, 0.30, *p* = .483), although the interventions did become more effective at increasing MVPA (b = 0.47, 95%CI = 0.12, 0.83, *p* = .013). Analyzing prompts from the second week of the intervention demonstrated a marginal effect on MVPA among parents (average causal effect = 2.73, 95%CI = −0.32, 5.79, *p* = .076, d = 0.33).

### Acceptability of the active family intervention

Descriptive statistics for the acceptability of the Active Family intervention are displayed in Table 4. Parents generally reported high agreement with the items on the implementation outcome scales assessing acceptability (e.g., I like the intervention), appropriateness (e.g., the intervention is suitable), and feasibility (e.g., the intervention is easy to use) (mean scores all > 3.91 on a 5-point scale). However, they reported lower scores for the intervention teaching them something new about getting their child active (*M* = 3.59 ± .83), helping their child to be more active (*M* = 3.45 ± .94), or helping themselves to be more active (*M* = 3.36 ± 1.02).

**Table 4:** Descriptive Statistics for Parental Acceptability of Active Family Intervention (*n = 22).*

	Mean (SD)^a^
**Acceptability Items**	
The Active Family intervention taught me something new about getting my child active	3.59 (.83)
The Active Family intervention helped get my child to be more active	3.45 (.94)
The Active Family intervention helped get me to be more active	3.36 (1.02)
**Implementation Items**	
***Acceptability Questions***	
The Active Family intervention meets my approval	3.95 (.88)
The Active Family intervention is appealing to me	4.05 (.71)
I like the Active Family intervention	4.05 (.82)
I welcome The Active Family intervention	4.18 (.72)
***Appropriateness Questions***	
The Active Family intervention seems fitting	3.95 (.82)
The Active Family intervention seems suitable	4.00 (.85)
The Active Family intervention seems applicable	3.91 (.90)
The Active Family intervention seems like a good match	3.82 (.94)
***Feasibility Questions***	
The Active Family intervention seems implementable	3.91 (.90)
The Active Family intervention seems possible	4.23 (.60)
The Active Family intervention seems doable	4.18 (.83)
The Active Family intervention seems easy to use	4.36 (.83)

^a^All items were rated on a 5-point Likert scale (*1 = Completely disagree to 5 = Completely agree*).

Parents’ perspectives of the Active Family intervention (through open-ended questions; see Table 5) revealed that parents valued receiving activity ideas that extended beyond those they would typically think of themselves and felt that the intervention made them more conscious and aware of their activity levels and how much time they are sedentary. Though, most parents reported that their PA usually occurred away from the home, and most prompts were designed for when they were at home, which did not always align with the family routine. Further, the text messages with activity prompts were sometimes at inconvenient times when it was not viable to be active (i.e., during mealtimes). With this, parents provided some suggestions for improvement including customized prompt times, activity suggestions for different contexts, as well as providing education on the importance of their children’s PA levels to motivate activity to happen. A final drawback to the intervention was around the mode of delivery with frequent texts, which made parents spend more time on their phones around their children.

**Table 5:** Parents’ Perspectives of the Active Family Intervention.

Deductive Framework Category	Inductive Themes	Example quotes from open-ended questions
**Likes**	Helpful activity ideas	*‘The activity suggestions were very creative; they aren’t things I would’ve thought of on my own. It was also nice to be reminded of doing activity, even if I didn’t do the ones suggested.’* *‘I liked the prompts with creative suggestions; they were helpful for coming up with ways to move. It can be tiring as a parent to always come up with ideas and activities.’*
	Increased awareness of activity and movement levels	*‘It definitely made me more aware of our activity levels and gave me new ideas of indoor ways to be active with my child.’* *‘Increased awareness of active vs sedentary time.’*
**Dislikes**	Intervention via text messages inadvertently promoted more parental screen engagement	*‘The delivery of notifications actually increased time I had my phone/was looking at my phone when I went to spend less time with my phone especially during the day around my kids.’* *‘I personally don’t like to have my phone with me around my child. So, I found I had it with me all the time and was waiting for the texts to come through. For some people, this wouldn’t be a problem and would prompt them to be more active and possibly present with their child.’*
	Home based activity suggestions misaligned with existing family activity routines	*‘Seemed to presume we could only increase activity levels at home. I found the prompts/activity ideas when home were geared to someone else’s living situation (i.e., we live in a small apartment with only one open space and usually need to discourage physical play there for fear of injuries, etc. We do our active play outside (multiple times daily) and creative play (pretend, art/craft, cooking, reading, board games etc) are home.’* *‘There were no options like when you are not at home (for example in the park).’*
	Inconvenient activity prompt timing	‘*Some reminders came at meal times’* *‘Also many times activities were suggested when my child was sitting down during meals or while I was helping get the older sibling ready for school etc.’* *‘Many times when I received a text, I wouldn’t be able to follow the suggestions because I was cooking/cleaning/taking care of my other baby/sitting down to eat.’*
	Adding to feelings of parental burden	*‘The little “encouragements” to “get active” at the end of the daily questionnaires were sometimes overwhelming and off-toned. For example, my daughter would be “sitting around” in the house after playing outside in 30°C, etc. It made me feel like I was not getting her enough exercises.’* *‘… I think more encouragement in the questions would have been helpful with prompts of saying “do this for 5 minutes” etc. Because at times when I felt overwhelmed it felt like another thing to think about whereas if I was reminded of the benefits of it, I might have been more willing to quickly try.’*
Improvements	Parental-focused educational support to help characterize child activity levels	*‘Maybe add a definition to parents about what being active really means. Does it mean running? Or just moving around during play?’* *‘I think some education around what constitutes as your child being active vs not active would have been helpful. For example, playing, cleaning, etc. And the amount of time…I think more encouragement in the questions would have been helpful with prompts of saying “do this for 5 minutes” etc. Because at times when I felt overwhelmed it felt like another thing to think about whereas if I was reminded of the benefits of it, I might have been more willing to quickly try.’*
	Activity suggestions for different contexts (i.e., not at home, no equipment available)	*‘I think there could have been more suggestions on quick ways to get active whether you are at home or not. I would have appreciated a list of ways to get active with your child that I could refer back to at times when we had a moment.’* *‘Activities that are require no preparation, and are suited to one child rather than a group would have been more helpful.’*
	Customized prompt times	*‘Also, if there was some opportunity to input your schedule (general wake-up times, unavailable times, bedtime, etc.) to customize when you get your prompts a bit, that would be really helpful!’* *‘Ask participants and implement do not disturb hours. Such as meal times and other organized non active activities such as crafts etc’* *‘I think the most valuable part of this concept would be real time notifications that deliver activity prompts after 30-50 minutes of inactivity based on a schedule that parents can pre-program to accommodate naps and bedtime.’* *‘…Or if you could schedule activity suggestions for times when you know your child often is sitting and you have time to do activities with them (e.g., mid-morning, instead of when getting ready for school, lunch time, cooking/eating dinner, etc)’*

## Discussion

This study aimed to examine the effectiveness and acceptability of the Active Family m-health intervention via a JITAI micro-randomized controlled trial on the PA and ST of young children and their parents. Results suggest that the intervention was largely acceptable to parents of young children but overall did not result in proximal changes in ST, LPA, or MVPA of children or their parents. However, over the 2-week intervention period, the intervention became more effective at increasing MVPA of parents. In addition, among children, the intervention was more effective at reducing ST when indoors (rather than outdoors), and increasing PA when children were just with their parents (and not also with other adults) and when the weather was pleasant (compared to too cold).

In general, these findings indicate that providing contextually tailored just-in-time activity suggestions to stay-at-home parents may not be sufficient to alter children’s movement behaviours. However, the results did demonstrate some promising findings on children’s movement when the prompts aligned with environmental and contextual facilitators. For example, the results showed that the intervention may be more effective when targeting children when they are at home inside, when the weather is pleasant, and when the parent and their child are not with other adults. These findings align with existing research evidence. For example, research shows that children accumulate most of their ST indoors [61]; therefore, prompts may help to interrupt or encourage movement in environments where children are more likely to be sedentary. Additionally, there is evidence supporting seasonal variations in children’s PA behaviours [62], demonstrating the role that weather can play in impacting children’s PA. These findings could be used to tailor future JITAI. For example, a weather application could be integrated into JITAI and GPS data could be used to provide more tailored and appropriate prompt content. Additional customization of the intervention, including both the prompts (e.g., specific to their circumstances) and the timing of the prompts, was recommended by participants. This may in turn mean that parents and children are more able to be receptive to the suggestions, and therefore, the interventions may be more likely to be effective at changing proximal behaviours. However, it is important to note that these analyses are exploratory, and significant moderation effects could reflect statistical noise. Further research is warranted to determine under what conditions JITAIs may be effective in parent-child dyads.

Parents stated that the Active Family intervention was largely acceptable, appropriate, and feasible, though did offer suggestions for improvement. This adds to a growing body of evidence demonstrating that JITAIs are considered acceptable across diverse samples [63]. Though, qualitative feedback revealed meaningful barriers (i.e., inconvenient timing of messages, misalignment with family/home routines) that may influence real-world uptake. Parents provided suggestions that could inform refinement of the intervention to reconcile the tension between acceptability and implementation in daily life. Suggestions concerned customized prompt times and activity suggestions for different contexts, as they sometimes misaligned with existing family routines. The regularity of prompts (7 times per day) and following prompting schedules that had allowed for complete randomization of timing of texts resulted in frequency and timing of the texts not always being convenient (e.g., texts at mealtimes). While this study used EMA intervention approaches [64], which relies on parents completing EMA assessments to report on availability and tailoring variables, the intervention could benefit from leveraging passive sensors to collect data in the future to reduce participant burden. For example, commercial activity trackers can be used to continuously track PA, ST, and sleep behaviours, and send prompts when an activity threshold is met (e.g., prolonged sedentary periods). This will help ensure that JITAIs are only sent to participants as needed based on their activity thresholds. Parents also suggested that pre-programming times when they are not available (i.e., more granularity for when unavailable such as mealtimes, bedtimes) to ensure they do not receive texts during this time may help with compliance. Additionally, some parents in the current study reported that receiving the intervention via text messages inadvertently promoted more screen time. This may be perceived as contradictory and counter-intuitive to the objective of the study given that the negative implications of screen use include reducing the amount of available time for behaviours beneficial to health (i.e., PA [65]).

In the current intervention, most activity suggestions centred on activities that could be done in the home. The reason for this was that families may be engaged in many other activities when away from the home (e.g., grocery shopping, going to appointments, visiting friends, etc), which would mean that they would be unable to complete an activity suggestion. Despite this, parents discussed ways to improve the intervention content, including expanding activity suggestions for different contexts, this included suggestions for when they are away from home (some parents noted all their children’s movement takes place outside of the home due to space limitations) and with various pieces of equipment. A co-design approach should be taken to design intervention content with parents to meet their needs. Considering ways to personalize the prompts at the participant level could be an approach to improve the effectiveness of activity suggestions. For example, if it is known that participants live in a dwelling without a yard, suggestions can be tailored to ensure that activity suggestions reflect this barrier. Generative artificial intelligence could be used to develop highly personalized content for individual participants to account for many different circumstances to help families become more active [66]. It is also plausible that at some intervention texts, children may have been engaging in sedentary behaviours, such as reading and crafts important for their development, and parents may not have been inclined to break up this type of ST [67]. Parents also discussed how they would like more general educational material related to the benefits and importance of PA for young children. Therefore, the JITAI could be augmented with static content which is accessible to parents throughout the intervention.

Results demonstrated that the intervention could have positive effects on parents’ PA. Specifically, results showed that the micro-interventions were effective at increasing proximal MVPA in the later part of the intervention period. While intervention content was not tailored specifically to parents, it did focus on getting their child active, primarily through adult-led physical activities. Interestingly, this is the opposite of what has been observed in other studies where JITAIs have targeted adult participants, where the proximal effects on PA reduce over the course of the study [44,45]. This could suggest that parents became more aware of their own behaviours over the course of the intervention, which then prompted them to change their own behaviour, or had begun to adjust to expecting activity texts throughout the day. Behaviour change theories such as the Theory of Planned Behaviour [68] suggest that intention is the most proximal determinant of behaviour change and has been shown to be a key predictor of MVPA in parents of young children participating in a PA intervention [69]. In particular, goal-setting, habits, and exercise identity predicted intention to behaviour change [69]. A meta-regression of m-health interventions suggested that including elements of goal setting or self-monitoring in intervention design are most effective at increasing PA [70]. Parents of young children in the Mini Movers intervention (which saw significant effects on young children’s screen time) stated that the goal-planning aspect of the intervention was useful and helped them stay on track [36]. Taken together, this could offer a potential explanation to our findings, in that the repeated prompts may have helped parents form or strengthen intentions and set goals regarding their own activity, which would take time to develop, and would explain the emerging effects by week two. However, it is unclear from this study whether these effects would be continued and sustained long-term. Future research should examine whether implementing goal-setting aspects within a JITAI m-health intervention to parents with young children would increase effectiveness, as well as employing longer term follow-ups to determine sustained effectiveness.

## Strengths and limitations

There are several strengths to this study. First, micro-randomized controlled trials using JITAIs are a novel methodology in the e/m-health intervention space for improving PA and movement behaviours and can be used to identify under what conditions m-health interventions are likely to be effective [71]. To our knowledge, this is the first study exploring the effectiveness and acceptability of an m-health intervention on the PA and ST of young children and their parents using this methodology. Through collecting both effectiveness and acceptability data, this study provides several actionable approaches to improving the implementation of JITAIs in young children and their parents moving forward. With this, having parents’ feedback on this small scale, first of its kind study, will not only improve child m-health interventions, but also, the study design and measurement schedule of future JITAIs. Further, the use of accelerometer derived PA and ST outcomes provides a robust measurement of behaviour over self-report, minimizing social desirability bias. The 60-minute window prior to and following a prompt to determine changes in activity levels was selected to be consistent with previous micro-randomized controlled trials of physical activity [45,46]. This period of time is also logical from a practical perspective as parents would need to initiate behaviour change in their child, and they may not have acted instantaneously upon receiving an activity suggestion. That being said, future research may wish to examine changes in activity levels over varying windows of time to determine if this has substantial implications of activity behaviours in an EMA intervention context. Nevertheless, there are several limitations to the current study that need to be considered. First, the study included a small sample with a limited number of participants with usable data (i.e., participants were available to receive a micro-intervention less regularly than anticipated). With this, while the sample included families with different ethnic backgrounds and spanning several provinces, participants were primarily female and predominantly of white ethnicity. While we intentionally targeted stay-at-home parents and their young children to ensure that parents and children spent a large amount of their time together to test the intervention, focusing solely on this population limits the generalisability of these findings. Future larger-scale studies should test in a more representative sample before generalising these findings to other samples. Secondly, though the use of accelerometry was a strength of this study, missing accelerometer data reduced the number of available data points to 65% for children and 61% for adults, which limits the sample for analysis and could have implications for internal validity, as available data may be representative of a bias sample. The activity suggestions were only tailored to a small number of contextual factors, due to this being a pilot study to test this approach, with relatively general categories. Future studies should consider other contextual factors, as well as other environments, such as child mood, future plans (e.g., whether the parent and child will be available for the entire 60 minutes after a prompt), and activities away from the home. As well, future studies may assess variables with greater granularity to improve the appropriateness of activity suggestions. While this was a pilot study to determine effectiveness and acceptability, these limitations must be considered. Intervention refinement considering parental feedback should guide a future large-scale study.

## Conclusion

The Active Family m-health intervention was considered acceptable, appropriate, and feasible. However, the micro-interventions did not significantly change proximal ST, LPA, or MVPA of young children. Though, the intervention did show promise for increasing parents’ MVPA over time and pointed to situations where contextually tailored activity suggestions may be effective for changing young children’s activity. Parents provided several suggestions to improve the intervention prior to future larger-scale testing.

## Supporting information

S1 File Ecological Momentary Assessment Questionnaire. (DOCX)
