# Supplementary material for: A pilot effectiveness study of a just-in-time micro-randomized controlled trial on the physical activity and sedentary time of young children and their parents: The active family m-health intervention
Source: PLoS One. 2026 Jan 16;21(1):e0340687. doi: 10.1371/journal.pone.0340687 (PMC12810825; doi:10.1371/journal.pone.0340687)
Supplement: S1 File — (DOCX) [file pone.0340687.s001.docx]

**S1 File**

**Ecological Momentary Assessment Questionnaire**

| **Section** | **Questions** |
| --- | --- |
| **Availability** | Are you and your child together right now?   - Yes - No   Is your child awake right now?   - Yes - No   Are you at home right now?   - Yes - No   What is your child doing right now?   - Sitting down - Standing around - Being active |
| **Location** | Are you inside or outside right now?   - Indoors - Outdoors |
| **Weather** | How would you describe the weather right now?   - Pleasant - Too cold - Too hot - Raining - Snowing |
| **People** | Who are you and your child with right now? (select all that apply)   - No one else - Other adults - Other children |
